# Initialization of quantum simulators by sympathetic cooling

**DOI:** 10.1126/sciadv.aaw9268

**Published:** 2020-03-06

**Authors:** Meghana Raghunandan, Fabian Wolf, Christian Ospelkaus, Piet O. Schmidt, Hendrik Weimer

**Affiliations:** 1 Institut für Theoretische Physik, Leibniz Universität Hannover, Appelstraβe 2, 30167 Hannover, Germany.; 2 QUEST Institut, Physikalisch-Technische Bundesanstalt, Bundesallee 100, 38116 Braunschweig, Germany.; 3 Institut für Quantenoptik, Leibniz Universität Hannover, Welfengarten 1, 30167 Hannover, Germany.

## Abstract

Simulating computationally intractable many-body problems on a quantum simulator holds great potential to deliver insights into physical, chemical, and biological systems. While the implementation of Hamiltonian dynamics within a quantum simulator has already been demonstrated in many experiments, the problem of initialization of quantum simulators to a suitable quantum state has hitherto remained mostly unsolved. Here, we show that already a single dissipatively driven auxiliary particle can efficiently prepare the quantum simulator in a low-energy state of largely arbitrary Hamiltonians. We demonstrate the scalability of our approach and show that it is robust against unwanted sources of decoherence. While our initialization protocol is largely independent of the physical realization of the simulation device, we provide an implementation example for a trapped ion quantum simulator.

## Teaser

We demonstrate the preparation of ground states of largely arbitrary Hamiltonians by sympathetic cooling with a single particle.

## INTRODUCTION

Quantum simulation is an emergent technology that can potentially solve important open problems related to high-temperature superconductivity, interacting quantum field theories, or many-body localization (*1*). While a series of experiments demonstrated the successful implementation of Hamiltonian dynamics within a quantum simulator (*2*–*14*), these works had the simulator initialized in an easily accessible state such as a product state. Consequently, adiabatic evolution from an initial Hamiltonian whose ground state can be prepared to the final Hamiltonian of interest has been used. However, this approach becomes challenging across quantum phase transitions, especially if the transition is of first order.

Our strategy to overcome this problem builds on the recent advances in using dissipative quantum systems to engineer interesting many-body states as the attractor states of such an open quantum many-body system (*15*–*25*). In the past, these dissipative state engineering schemes have been limited to ground states of stabilizer or frustration-free Hamiltonians (*16*, *17*, *26*, *27*), whose ground state can be found by performing local optimizations alone. Unfortunately, almost all many-body Hamiltonians of interest lie outside this class, requiring generalization of the dissipative state preparation procedure.

Here, we present a previously unexplored paradigm for the dissipative initialization of a quantum simulator. We consider a coupling of the many-body system performing the quantum simulation to an auxiliary particle that is dissipatively driven. Crucially, the energy splitting within the auxiliary particle is chosen such that it becomes resonant with the many-body excitation gap of the system of interest, i.e., the difference of the ground-state energy and the energy of the first excited state. Under such a resonance condition, the energy of the quantum simulator is efficiently transferred to the auxiliary particle such that the former is cooled sympathetically (*23*, *25*). Although this setup is only resonant at a single energy, the density of states increases exponentially with energy, resulting in the lowest-lying excitations being the bottleneck for fast ground-state preparation (see the Supplementary Materials for details). While the value of the many-body excitation gap is usually unknown before performing the simulation, we demonstrate that the gap can actually be determined from the quantum simulation data in a spectroscopic measurement. Hence, the dissipative initialization process provides important information about the many-body system of interest at the same time. Notably, we show that the cooling by a single auxiliary particle is efficient, and it is especially robust against unwanted noise processes occurring in the quantum simulator.

**Fig. 1:**
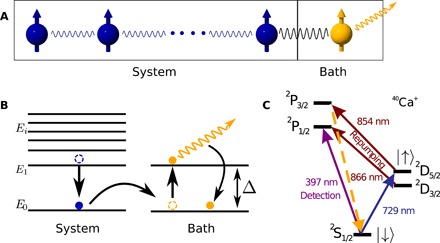
Sympathetic cooling of a quantum simulator. (**A**) A system of *N* spins performing the quantum simulation is interacting with an additional bath spin that is dissipatively driven. (**B**) Sketch of the energy level structure showing resonant energy transport between the system and the bath, after which the bath spin is dissipatively pumped into its ground state. (**C**) Level scheme for the implementation with trapped ^40^Ca^+^ ions.

To be explicit, we consider different paradigmatic one-dimensional (1D) spin 1/2 many-body systems coupled to a single dissipatively driven auxiliary bath spin (see Fig. 1). This setup can be readily generalized to bosonic or fermionic many-body systems with a larger local Hilbert space, to settings incorporating several bath particles, and to higher spatial dimensions. In the following, we assume a 1D chain of *N* spins governed by the Hamiltonian *H*_sys_. One boundary spin of the system is coupled to the auxiliary bath spin via an interaction Hamiltonian of the form $H_{\text{int}}=g_{\text{sb}}\sum_{x,y,z}f_{i}\sigma_{i}^{\left(N\right)}\sigma_{i}^{\left(b\right)}$, where *g*_sb_ is the strength of the system-bath interaction and the σ*_i_* refer to the Pauli matrices. The exact values of the dimensionless parameters *f_i_* are not particularly important. In the models studied here, we find that it is either favorable to choose them roughly equal or have one dominant contribution. In addition, to avoid any symmetries in the interaction preventing the cooling of certain degrees of freedom, it is beneficial to assign slightly different values to them.

The Hamiltonian of the bath spin *H*_bath_ is given by $H_{b}=(\Delta /2)\sigma_{z}^{\left(b\right)}$. The dissipation channel acting on the bath spins performs dissipative spin flips from the up spin state to the down spin state occurring with a rate γ. Then, the total dynamics is described by a quantum master equation in Lindblad form

$$\frac{d}{\mathrm{dt}}\rho =-\frac{i}{\hbar }[H,\rho ]+\gamma (\sigma_{-}^{\left(b\right)}{\rho \sigma }_{+}^{\left(b\right)}-\frac{1}{2}\{\sigma_{+}^{\left(b\right)}\sigma_{-}^{\left(b\right)},\rho \})$$ (1)

where *H* = *H*_sys_ + *H*_bath_ + *H*_int_ is the total Hamiltonian of the *N* + 1 spin system (*28*).

We would like to stress that such a setup imposes only modest requirements for an experimental implementation, which works equally well for both analog and digital quantum simulators. In particular, we note that our setup does not require control over individual particles of the quantum simulator. In our case, it is sufficient to merely be able to control the bath particle independently of the rest of the system. In addition, the dissipative dynamics can be induced by measuring the spin state of the bath spin followed by a spin flip conditional on measuring the spin in the up state. In Materials and Methods, we give a detailed implementation guide for a trapped ion quantum simulator.

## RESULTS

### Ising chain in a transverse field

As the first paradigmatic model, we consider the Ising model in a transverse field, given by the Hamiltonian

$$H_{\text{sys}}=g\sum_{i=1}^{N}\sigma_{z}^{\left(i\right)}-J\sum_{i=1}^{N-1}\sigma_{x}^{\left(i\right)}⊗\sigma_{x}^{\left(i+1\right)}$$ (2)

where *g* is the strength of the transverse field, and *J* is the coupling constant for the Ising interaction. As the Pauli matrices do not commute with each other, it is impossible to minimize the interaction terms and the magnetic field term at the same time, meaning that already this simple model lies outside of the class of frustration-free Hamiltonians. The transverse field Ising model is known to undergo a quantum phase transition at *g* = *J* from a paramagnetic phase (*g* > *J*) to a ferromagnetic phase (*g* < *J*) (*29*). In the following, we will set the energy splitting of the bath spin Δ to be identical to the many-body gap Δ*E* = *E*_1_ − *E*_0_ of the transverse field Ising model, where *E*_0_ (*E*_1_) is the energy of the ground state (first excited state). In the ferromagnetic phase, the ground state becomes doubly degenerate for large system sizes. Because we are not interested in cooling into a particular ground state, *E*_1_ refers to the first excited state above the ground-state manifold. Below, we will demonstrate that choosing the bath spin splitting as Δ = Δ*E* leads to optimal cooling, and we will show how to extract the (a priori unknown) energy gap Δ*E* from the quantum simulation results.

**Fig. 2:**
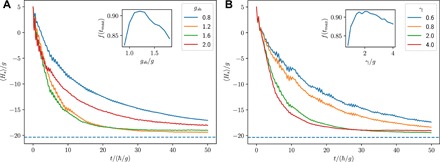
Sympathetic cooling of the transverse field Ising model in the ferromagnetic phase (*J/g* = 5, *N* = 5, *f_x, y, z_* = {1,1.1,0.9}). The speed of the cooling dynamics and the final energy of the system depend on the system-bath coupling *g*_sb_ for γ/*g* = 1.9 (**A**) and the dissipation rate γ for *g*_sb_/*g* = 1.15 (**B**). The ground-state energy is indicated by the dashed line. The insets show that the ground state can be prepared with greater than 90% fidelity.

Let us now analyze the cooling performance of the setup by tracking the system energy 〈*H*_sys_〉 of the transverse field Ising model in wave-function Monte Carlo simulations of *N* = 5 spins, initially in the experimentally accessible state of all spins pointing up. Figure 2 shows that the energy of the system decreases rapidly and finally approaches a value that is close to the numerically calculated ground-state energy. The cooling performance depends on the choice of the system-bath coupling *g*_sb_ and the dissipation rate γ. In the following, we assume that the time available for the cooling remains fixed. Then, if *g*_sb_ is too small, the cooling dynamics is very slow. On the other hand, if *g*_sb_ is too large, then the system and the bath spin will become strongly entangled, and the cooling performance is reduced. Similarly, if γ is too small, then the cooling is slowed down in the same way, while a too large value of γ will lead to a quantum Zeno suppression of the energy transfer required for the cooling process. Hence, there should be an optimal choice for *g*_sb_ and γ, which leads to a minimum in energy within the available time.

To find this optimal choice, we use a model-independent quantity to measure the cooling performance. For this, we calculate the fidelity of the state of the system with respect to the ground-state manifold of the transverse field Ising model. The fidelity *f* is given by

$$f=\langle \Pi_{g}\rangle =\text{Tr}\{\rho (t)\Pi_{g}\}$$ (3)

where $\Pi_{g}=\sum_{i}|\psi_{0}^{i}\rangle \langle \psi_{0}^{i}|$ is the sum of the projectors onto the ground states (*30*). As the inset of Fig. 2 (A and B) shows, the ground state can be prepared with more than 90% fidelity for the optimal choice of *g*_sb_ = 1.15 *g* and γ = 1.9 *g*.

We can also relate the fidelity *f* to the system energy 〈*H*_sys_〉. For this, we introduce a dimensionless excitation energy ε, measured in units of the many-body gap Δ*E*, i.e.

$$\varepsilon =\frac{\langle H_{\text{sys}}\rangle -E_{0}}{\Delta E}$$ (4)

In the low-energy limit ε ≪ 1 and assuming that the excitation energy is mostly concentrated in low-energy excitations, ε is related to the fidelity according to ε = 1 − *f*.

We have also checked that our cooling procedure works independently of the choice of *J*/*g*, i.e., both in the ferromagnetic phase and in the paramagnet, as well as independently of the initial state (see the Supplementary Materials for details). Even in the critical regime (*J*/*g* ∼ 1), where the many-body gap is closing, we observe a similar cooling performance. To substantiate this point and also to demonstrate that our cooling protocol is not limited to a particular model, we turn to the especially challenging case of a critical Heisenberg chain in the following section.

### Antiferromagnetic Heisenberg model

As a second paradigmatic quantum many-body model, we investigate the antiferromagnetic Heisenberg chain, given by the system Hamiltonian

$$H_{\text{sys}}=J\sum_{i=1}^{N-1}\sum_{j=x,y,z}\sigma_{j}^{\left(i\right)}⊗\sigma_{j}^{\left(i+1\right)}$$ (5)

This model exhibits an *SU*(*2*) symmetry and serves as the critical point of a Kosterlitz-Thouless transition when the strength of the σ*_z_*σ*_z_* interaction is varied (*31*). As the many-body gap vanishes in the thermodynamic limit, this model represents a particularly challenging case for our cooling protocol. In addition, the ground state at the critical point is highly entangled (*32*); hence, we also test the capability of our cooling protocol to prepare entangled quantum many-body states.

**Fig. 3:**
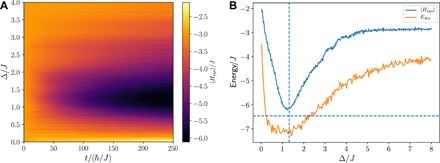
Sympathetic cooling of the antiferromagnetic Heisenberg model (*N* = 4, *g*_sb_/*J* = 0.2, γ/*J* = 0.6, *f_x,y,z_* = {0.4,2.3,0.3}). (**A**) The efficiency of the cooling procedure depends on the choice of the bath spin splitting Δ. (**B**) The optimal cooling leading to the lowest system energy 〈*H*_sys_〉 corresponds to setting Δ to the many-body gap Δ*E* (vertical dashed line). The same minimum is observed when measuring the energy *E*_dis_ that is being dissipated during the cooling process. The ground-state energy is indicated by the horizontal dashed line.

The antiferromagnetic Heisenberg model adds one minor complication concerning its ground state preparation compared with the Ising model. Because of an approximate symmetry conserving certain spin-wave excitations, the ground-state cooling performance is limited when the system-bath coupling is restricted to the last spin of the chain. We resolve this issue by an additional system-bath coupling of strength *g*_sb_/2 to the second last spin of the chain. Figure 3 shows the cooling performance in terms of the system energy 〈*H*_sys_〉, with an initial state of spins pointing up and down alternately, as a function of the splitting of the bath spin Δ. As in the case of the transverse field Ising model, 〈*H*_sys_〉 decreases rapidly and reaches a final value that is close to the ground-state energy *E*_0_. In addition, the cooling is optimal when Δ is chosen to be identical to the many-body gap Δ*E* (*f* = 0.9). Hence, experimentally measuring *H*_sys_ as a function of Δ allows one to obtain the value of the many-body gap Δ*E*, which in itself is an important quantity to understand a quantum many-body system. We also find the final state to be highly entangled (see the Supplementary Materials for details).

However, on many quantum simulation architectures, it might be difficult to experimentally measure the system energy *H*_sys_, as this will typically require one to perform tomography on all the operators that appear in the system Hamiltonian. Further challenges arise in architectures where not all coupling constants in the Hamiltonian can be perfectly controlled, leading to additional uncertainties in the estimated value of Δ*E*.

Fortunately, it is possible to obtain Δ*E* by measuring only the bath spin. The key idea is to measure the energy *E*_dis_ that is dissipated during the cooling dynamics. Crucially, this energy is related to the number of quantum jumps *N*_jump_ by the relation *E*_dis_ = *N*_jump_Δ, as a quantum jump will lower the energy of the bath spin by Δ. We note that there are two different ways to obtain *N*_jump_. First, one can directly count the number of quantum jumps, e.g., by counting the number of emitted photons, if the dissipative flip of the bath spin is realized by a spontaneous emission event. In many setups, however, collecting each emitted photon with high probability might be too challenging. However, as a second method, one can also obtain *N*_jump_ via the integrated probability to find the bath spin in the up state according to

$$N_{\text{jump}}=\gamma \int_{0}^{t_{p}}\text{Tr}\{\sigma_{+}^{\left(b\right)}\sigma_{-}^{\left(b\right)}\rho (t)\}\;\mathrm{dt}$$ (6)

where *t*_p_ is the total preparation time. As shown in Fig. 3, the minimum of *E*_dis_ is almost identical to the minimum in *H*_sys_, corresponding to the case where the splitting of the bath spin Δ is identical to the many-body gap Δ*E*. We note that if the system-bath coupling *g*_sb_ or the dissipation rate γ is chosen too large, then the difference between the minima in 〈*H*_sys_〉 and *E*_dis_ becomes appreciably larger. We also observe that *E*_dis_ is slightly larger in magnitude than the system energy; this can be attributed to the fact that even in the limit of large times, a finite probability for quantum jumps remains, as the ground state of the system Hamiltonian is not a perfect dark state of the quantum master equation (*33*) due to the finite system-bath coupling *g*_sb_. These additional jumps can also happen for nonoptimal values of Δ, leading to a broadening of the dissipated energy *E*_dis_ in Fig. 3B compared with the system energy 〈*H*_sys_〉.

### Efficiency of the cooling protocol

For any quantum-state preparation protocol, it is crucial to determine how its properties behave when the size of the system is increased. A protocol is called efficient when the resources required (i.e., the preparation time) grow at most polynomially with the system size. To determine the scaling with system size in an unbiased way, we compute the preparation time *t*_p_ that is required to cool the system down to a fixed dimensionless energy ε, while the system-bath coupling *g*_sb_ and the dissipation rate γ are chosen such that the cooling is optimal. Within our numerical simulations, we use a standard nonlinear optimization scheme (see Materials and Methods for details). In an actual quantum simulator, one can use a hybrid algorithm in which the energies measured on the quantum device are fed back into the classical optimization algorithm (*34*).

**Fig. 4:**
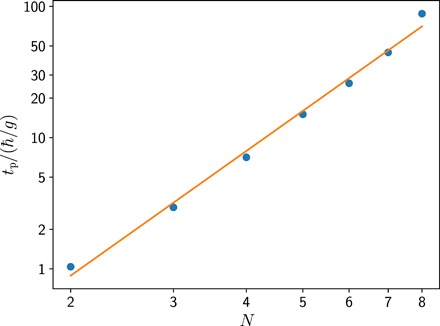
Scalability of the cooling protocol. The preparation time *t*_p_ to reach a final dimensionless energy of ε = 0.2 grows linearly on a log-log scale, i.e., *t*_p_ ∼ *N*^α^. The solid line is a fit to the data according to α = 3.1 ± 0.1.

Figure 4 shows the scaling behavior of *t*_p_ for the transverse field Ising model. Although the system is cooled across the phase transition into the ferromagnet, the preparation time grows only polynomially with the system size. The same scaling is also observed for the antiferromagnetic Heisenberg model (see the Supplementary Materials for details). This scaling behavior underlines that our cooling procedure is already scalable when using only a single bath spin. As the number of particles is often a scarce resource in a quantum simulator, the required minimal overhead for the initialization allows us to use almost all of the particles for the actual quantum simulation.

### Performance under decoherence

So far, the only source of decoherence in our considerations stems from the dissipative flips of the bath spin. However, in most quantum simulation architectures, there will also be unwanted decoherence processes in the system performing the quantum simulation. Therefore, it is crucial to determine the consequences of this additional decoherence on the performance of our cooling protocol.

As an additional source of decoherence, we consider σ*_z_* spin flips in the quantum simulation of the transverse field Ising model, applied with a rate κ to all *N* spins of the quantum simulator. In the ferromagnetic phase, such a spin flip will create two neighboring domain-wall excitations; i.e., when applied to the ground state, the dimensionless energy will approximately increase to ε ≈ 2. This type of decoherence represents a worst case scenario of all local decoherence processes. Hence, we expect that this scenario is quite generic and that our findings should also apply to other many-body models.

To analyze the consequences of these additional decoherence channels, we consider the quantity κ*t*_p_, which is essentially the probability of any spin to undergo a decoherence event during the preparation time. Then, tracking how the energy ε behaves as a function of κ*t*_p_ allows us to assess the robustness of our cooling protocol under additional decoherence.

**Fig. 5:**
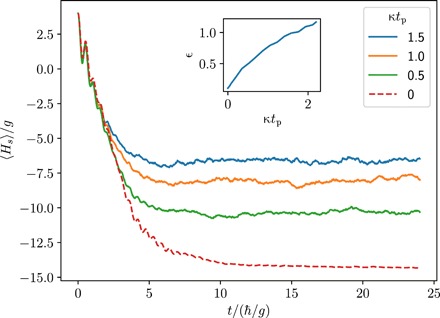
Cooling performance in the presence of decoherence in the quantum simulator for the transverse field Ising chain (*J/g* = 5, *N* = 4). The inset shows the dimensionless energy ε as a function of the product κ*t*_p_, where *t*_p_ was taken from the dynamics without decoherence corresponding to a ground-state preparation fidelity of *f* = 0.9 (dashed line).

Figure 5 shows the system energy for different decoherence rates, from which the behavior of ε is calculated. Crucially, we find that the system contains one excitation, ε ≈ 1 at a value of κ*t*_p_ ≈ 2. This means that the system picks up one excitation when on average all the spins have undergone a decoherence event. This is in stark contrast to the scaling observed in adiabatic state preparation protocols, where the error probability is typically given by the probability that a single spin undergoes a decoherence event, i.e., proportional to *N*κ*t*_p_ (*35*). This improved robustness against decoherence can be attributed to the fact that our state preparation protocol itself is dissipative and therefore can self-correct decoherence events.

### Experimental realization

The proposed initialization protocol can be implemented in a trapped ion system with state-of-the-art technology, e.g., by confining a 1D ion string in a linear Paul trap. Here, we propose an implementation with ^40^Ca^+^ ions in a setup similar to the one described in (*36*). The spin states are encoded in the optical qubit, ∣↓〉 = ∣*S*_1/2_, *m* = + 1/2〉 and ∣↑〉 = ∣*D*_5/2_, *m* = +5/2〉 (see Fig. 1C) with an energy splitting of ℏω_0_, coherently manipulated by radial laser beams; e.g., the rightmost ion serves as the bath spin (index *b*), while its laser-induced coupling to the neighboring ion (index *s*) implements the system-bath coupling. The bath ion can be isolated from the system interaction by shelving the population to an auxiliary state ∣aux〉*_b_* = ∣*D*_5/2_, *m* = −5/2〉*_b_* with a laser beam addressing only the bath ion. An experimental realization requires the implementation of the system and system-bath Hamiltonians. For simplicity, we suggest to implement *H*_sys_ and *H*_sb_ in an interleaved fashion by trotterizing the total interaction (*6*, *37*).

In trapped ion systems, *H*_sys_ for the transverse field Ising model (*5*) has been realized with up to 53 qubits (*12*). For this purpose, a global bichromatic laser beam with frequency ω_0_ ± δ implements a gate operation by coupling to all radial modes. If δ is larger than the center-of-mass mode frequency, then the resulting spin-spin coupling coefficient shows a power law scaling *J*_*i*, *j*_ ∝ 1/∣*i* − *j*∣^α^ (*38*), where α can be varied between 0 and 3 by changing the radial confinement. Implementation of the Heisenberg model is possible by interleaving the spin-spin coupling gates with single-qubit rotations performing a basis change from σ*_x_* to σ*_y_* and σ*_z_*.

We propose to implement *H*_sb_ with a separate laser that provides single ion addressing for the bath spin and the neighboring system spin. A Mølmer-Sørensen gate (*39*, *40*) on the radial motional modes bridges two different energy gaps, ω_s_ and ω_0_, similar to a two-species gate (*41*), and provides a $\sigma_{x}^{\left(N\right)}\sigma_{x}^{\left(b\right)}$-type coupling of the spins. For the bath spin, the laser frequencies will be ω_0_ ± δ, and for the system spin will be ω_s_ ± δ with ω_s_ = Δ*E*/ℏ for optimal cooling. Tuning the latter frequency corresponds to searching for the resonance condition described in the main text. Again, $\sigma_{x}^{\left(N\right)}\sigma_{x}^{\left(b\right)}$ gates interleaved with single-qubit rotations on both ions implement $\sigma_{x}^{\left(N\right)}\sigma_{x}^{\left(b\right)}$, $\sigma_{y}^{\left(N\right)}\sigma_{y}^{\left(b\right)}$, and $\sigma_{z}^{\left(N\right)}\sigma_{z}^{\left(b\right)}$. The coupling between the bath spin and the second last spin for the Heisenberg model can be realized by extending the addressing laser to the second last spin such that the power law of the system-bath interaction has an exponent α_sb_ ∼ 1. This comes at the expense of an additional interaction between the last and the second-last spins of the system, which is appreciably weaker than *J* and can therefore be neglected. This additional coupling may also be canceled using an additional addressing laser.

Assuming Δ*E* is already known, repumping from ∣↑〉*_b_* to P_3/2_ and a subsequent spontaneous decay to ∣↓〉*_b_* on the bath ion can be used to provide a channel for dissipation. The strength of dissipation, γ, within the trotterized scheme can be adjusted by the repumping laser intensity, i.e., the repumping probability during each Trotter cycle. For determination of Δ*E* by recording *N*_jump_, every scattered photon during the repump process has to be detected. This is accomplished by an electron shelving scheme in which the population in ∣↓〉*_b_* is hidden in state ∣aux〉*_b_* and a potentially scattered photon bringing the bath ion from ∣↑〉*_b_* to ∣↓〉*_b_* is detected by measuring fluorescence on the ∣↓〉*_b_*(S_1/2_)-to-P_1/2_ transition. To avoid a perturbation of the system spins, the detection laser has to be tightly focused onto the bath ion.

To be more specific, we assume 5^40^Ca^+^ ions in a linear chain with single ion axial and radial trapping frequencies of ω*_z_* = 2π × 0.15 MHz and ω*_r_* = 2π × 0.5 MHz, respectively (*36*). With a resonant Rabi frequency of 2π × 125 kHz for all ions, *J*_*i*, *j*_ ranges between 2π × 2.7 and 2π × 1.2 kHz for a detuning of δ − ω_r_ ≈ 2π × 10.5 kHz, while the largest Lamb-Dicke parameter is given by η_max_ = 0.128. For these parameters, the spacing between the bath spin and the nearest system spin of around 14 μm is sufficiently large to provide a factor of 10^−7^ suppression of the scattering rate for the electron shelving detection on the neighboring ion for a beam focused to 2.6 μm on the bath ion.

**Fig. 6:**
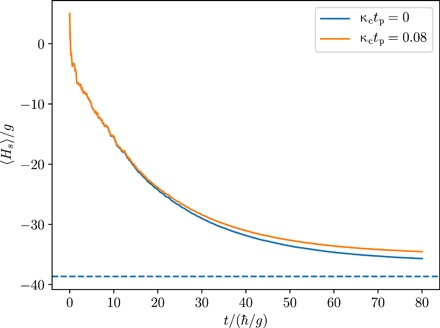
Cooling performance of an Ising-like chain of 5 + 1 ions of *t*_p_ = 80ℏ/*g* = 24s. The blue line shows the dynamics in the decoherence-free case resulting in a fidelity of *f* = 0.92, while the orange line indicates the dynamics under a collective decoherence mechanism with rate κ_c_ = 3.3Hz, resulting in *f* = 0.89. The dashed line indicates the ground-state energy of the system.

In such a setup, the dominant decoherence mechanism is arising from global magnetic field fluctuations. This process can be expressed in terms of a jump operator of the form $c\prime =\sqrt{\kappa_{c}}\sum_{i}\sigma_{z}^{\left(i\right)}$, assuming a decoherence rate of κ*_c_* = 3.3 Hz (*42*). Figure 6 shows the cooling of such a system to an optimized ground-state fidelity of *f* = 0.92 in the decoherence-free case, while the presence of decoherence leads to a fidelity of *f* = 0.89.

An alternative to single ion addressing is to use another isotope for the bath ion, such as ^44^Ca^+^. The large isotope shifts of 850 MHz on the S_1/2_-P_1/2_ transition and 5.3 GHz on the qubit transition (*43*, *44*) will appreciably relax the focusing requirements at the expense of having to achieve an appropriately ordered ion crystal (*45*).

## DISCUSSION

Here, we demonstrated how adding a dissipatively driven auxiliary particle can sympathetically cool a quantum simulator into low-energy states. Our approach is efficient even when using only a single bath spin, and it exhibits strong robustness against unwanted decoherence occurring in the quantum simulator. Future directions include investigating the scaling behavior when optimally varying the coupling constants of the bath in time and when adding multiple bath spins. In the latter case, it will also be of interest to choose different splittings of the bath spins, allowing engineering of tailored bath spectral functions for the quantum simulator.

## MATERIALS AND METHODS

### Numerical simulations

All numerical simulations were performed using a wave-function Monte Carlo approach provided by the QuTiP library (*46*), extended to a massively parallelized version (*47*). Results were obtained by averaging over 1000 Monte Carlo trajectories. We note that we are interested in the long time limit of a weakly dissipative system, i.e., a regime where tensor network algorithms are breaking down (*48*). Numerical optimization of the coupling constants *g*_sb_ and γ was carried out using a Nelder-Mead algorithm. We typically obtain convergence within approximately 50 runs of the simulation, which does not appreciably depend on the size of the system.

## Supplementary Material

aaw9268_SM.pdf

## SUPPLEMENTARY MATERIALS

Supplementary material for this article is available at http://advances.sciencemag.org/cgi/content/full/6/10/eaaw9268/DC1 Section S1. Energy-level representation of the cooling protocol Section S2. Cooling in the paramagnetic and the critical regime of the Ising model Section S3. Dependence of the cooling performance on the initial state Section S4. Efficiency of the cooling protocol for the Heisenberg model Section S5. Entanglement measure for the ground-state cooling of the Heisenberg model Fig. S1. Possible paths via which an excitation can be cooled down to the ground state. Fig. S2. Cooling dynamics in different regimes. Fig. S3. Cooling performance of the transverse field Ising model in the ferromagnetic phase for various initial states. Fig. S4. Scalability of the protocol for the antiferromagnetic Heisenberg model. Fig. S5. Negativity as a measure of entanglement of the prepared states in time for a system of *N* = 6 spins. References (*49*–*52*)
