# Supplementary material for: Initialization of quantum simulators by sympathetic cooling
Source: Sci Adv. 2020 Mar 6;6(10):eaaw9268. doi: 10.1126/sciadv.aaw9268 (PMC7060053; doi:10.1126/sciadv.aaw9268)
Supplement: aaw9268_SM.pdf [file aaw9268_SM.pdf]

Science Advances
AAAS

advances.sciencemag.org/cgi/content/full/6/10/eaaw9268/DC1

Supplementary Materials for

Initialization of quantum simulators by sympathetic cooling

Meghana Raghunandan*, Fabian Wolf, Christian Ospelkaus, Piet O. Schmidt, Hendrik Weimer

*Corresponding author. Email: meghana.raghunandan@itp.uni-hannover.de

Published 6 March 2020, *Sci. Adv.* **6**, eaaw9268 (2020)
DOI: 10.1126/sciadv.aaw9268

This PDF file includes:

Section S1. Energy-level representation of the cooling protocol
Section S2. Cooling in the paramagnetic and the critical regime of the Ising model
Section S3. Dependence of the cooling performance on the initial state
Section S4. Efficiency of the cooling protocol for the Heisenberg model
Section S5. Entanglement measure for the ground-state cooling of the Heisenberg model
Fig. S1. Possible paths via which an excitation can be cooled down to the ground state.
Fig. S2. Cooling dynamics in different regimes.
Fig. S3. Cooling performance of the transverse field Ising model in the ferromagnetic phase for various initial states.
Fig. S4. Scalability of the protocol for the antiferromagnetic Heisenberg model.
Fig. S5. Negativity as a measure of entanglement of the prepared states in time for a system of $N = 6$ spins.
References (*49–52*)

Section S1. Energy-level representation of the cooling protocol

The physical mechanism behind our cooling protocol can be understood by looking at the transitions between the energy levels E_i of the many-body Hamiltonian H_{sys}. In a very simplified picture, we assume that a cooling transition is possible when its energy difference $E_i - E_j$ is within the energy window provided by the bath spin, whose splitting Δ is broadened by the decay rate γ. Fig. S1 shows all possible transitions between energy levels with energy differences of $\Delta \pm \gamma$. After the energy is transferred from the many-body system to the bath spin, it can be dissipatively removed. After many such processes, the system is cooled down to its ground state. As seen from Fig. S1, there are multiple possible paths for cooling of the excited states, which enables high-fidelity ground state preparation. Note that the system-bath interaction explicitly breaks any possible symmetry of the system Hamiltonian, therefore also symmetry-breaking cooling transitions are possible.

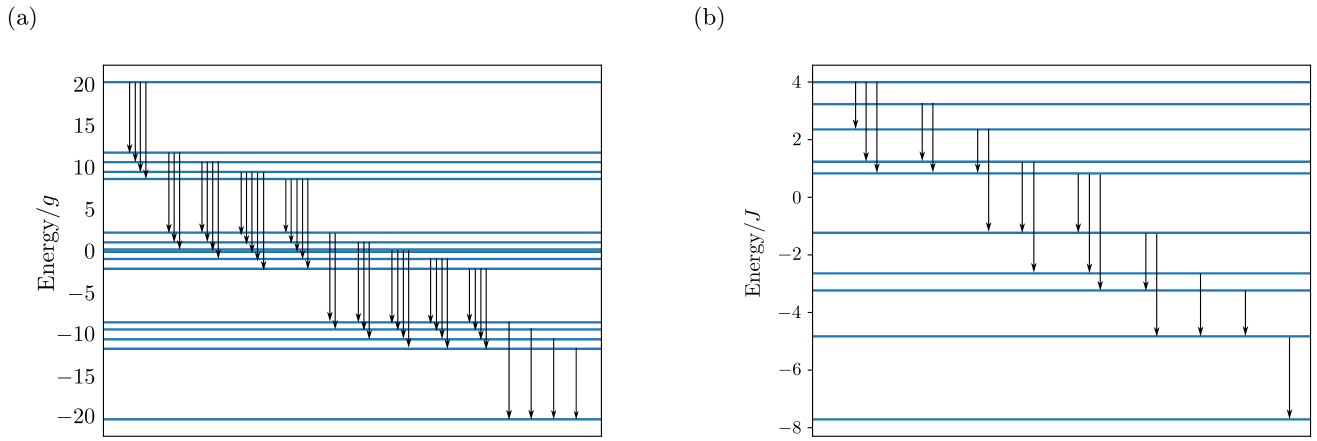

Fig. S1. Possible paths via which an excitation can be cooled down to the ground state. Each black arrow corresponds to an energy difference $\Delta - \gamma \leq E_i - E_j \leq \Delta + \gamma$. Each cooling step leads to a reduction of the energy of the system, eventually reaching the ground state. The energy levels are shown for (a) the Ising model ($N = 5$, $J/g = 5$, $\gamma/g = 3.5$) and (b) the Heisenberg model ($N = 5$, $\gamma/J = 1.26$).

Section S2. Cooling in the paramagnetic and the critical regime of the Ising model

Here, we check the performance of our cooling protocol in the paramagnetic ($g \gg J$) and the critical ($g \sim J$) regimes. Fig. S2 (a) and (b) show the cooling of an Ising chain with $N = 5$ spins in the paramagnetic phase whereas Fig S2 (c) and (d) show the cooling in the critical regime. We observe the existence of optimal values of the parameters, g_{sb} and γ for maximum cooling similar to the case of the ferromagnetic Ising model. Note that due to finite size scaling, the critical regime for a system of $N = 5$ spins is not at $J/g = 1$ but rather at $J/g = 1.4$ which we have determined using the peak of the magnetic susceptibility.

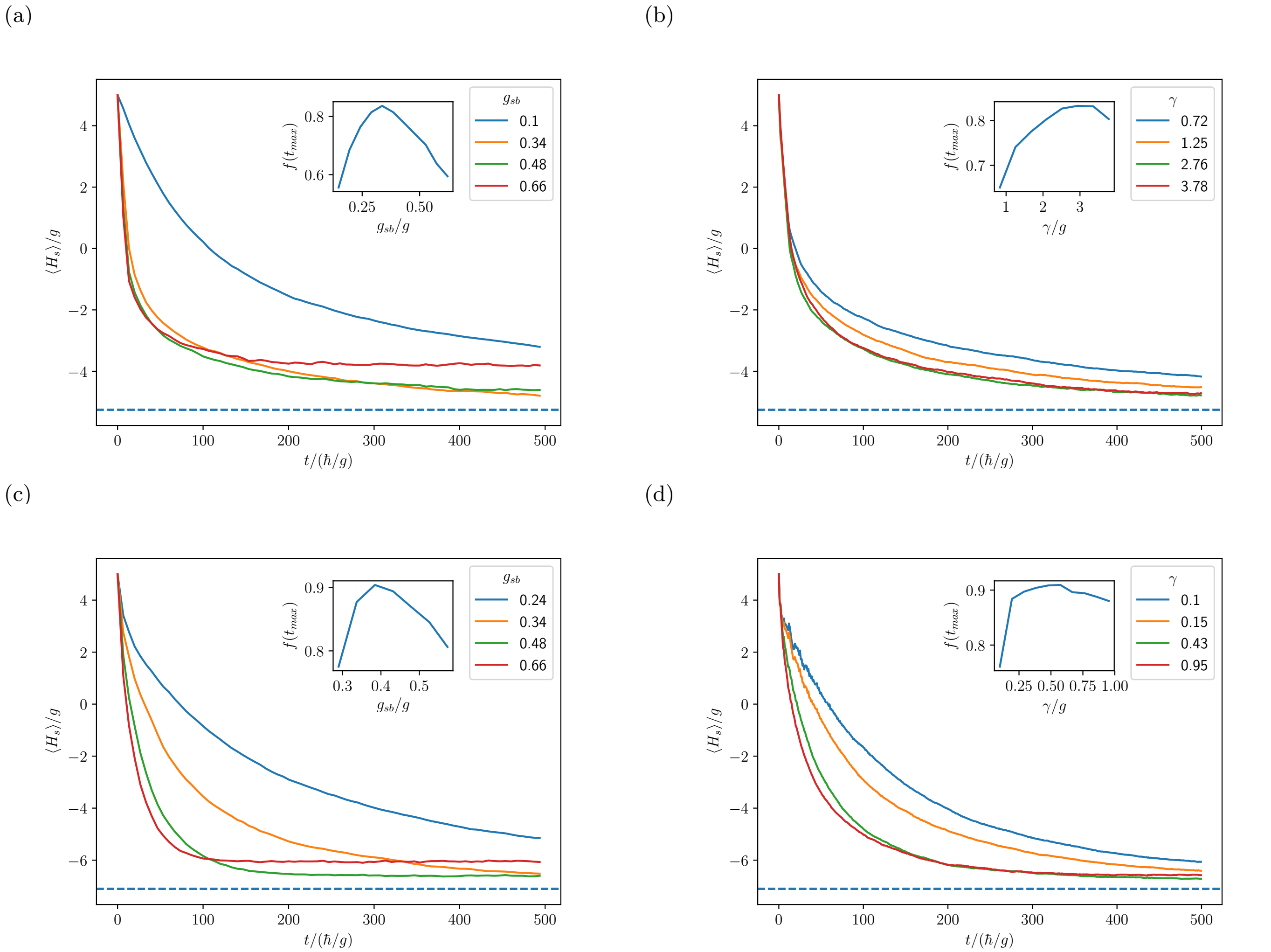

Fig. S2. Cooling dynamics in different regimes. Sympathetic cooling of the transverse Ising model ($N = 5$, $f_{x,y,z} = \{1, 1.1, 0.9\}$) in the paramagnetic phase ($J/g = 0.2$) (a-b) and in the critical regime ($J/g = 1.4$) (c-d).

Section S3. Dependence of the cooling performance on the initial state

While it is convenient to use an experimentally accessible state as the initial state for the cooling protocol, one may ask whether the cooling performance depends on the choice of the initial state. We investigate this dependence by choosing several product states with random configurations of up and down spins. In Fig. S3, we show the dynamics of the cooling for the transverse field Ising model. We see that the initial energies differ significantly, while the overall dynamics remains very similar. This picture is consistent with the cooling timescale being a property of the combined system-bath Liouvillian that does not depend on the state of the system.

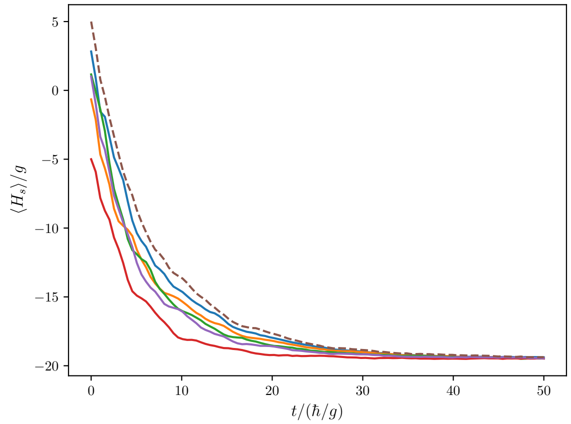

Fig. S3. Cooling performance of the transverse field Ising model in the ferromagnetic phase for various initial states. The dashed line corresponds to the case of all spins initially pointing up ($N = 5$, $J/g = 5$, $g_{sb}/g = 1.15$, $\gamma/g = 1.9$, $f_{x,y,z} = \{1, 1.1, 0.9\}$).

Section S4. Efficiency of the cooling protocol for the Heisenberg model

In the same way as for the Ising model, we determine the scaling of the preparation time t_p with the system size N for the antiferromagnetic Heisenberg model. Within spin-wave theory [49], one can see that the model exhibits a particular symmetry that makes cooling slightly more challenging than for the Ising model. The reason is a parity symmetry that arises when partitioning the model into two sublattices when constructing the spin-wave theory. Hence, it is possible that some excitations cannot be cooled when the bath spin is coupled to only the last site (and hence only to one of the two sublattices). Coupling the bath spin to the second-last site as well (here, we choose a coupling strength of $g_{sb}/2$) resolves the problem, i.e., $H_{int} = g_{sb} \sum_{x,y,z} f_i \sigma_i^{(N)} \sigma_i^{(b)} + \frac{g_{sb}}{2} \sum_{x,y,z} f_i \sigma_i^{(N-1)} \sigma_i^{(b)}$.

Fig. S4 shows that the optimal preparation time t_p for the antiferromagnetic Heisenberg model scales polynomially with the system size N. The smaller preparation times for odd system sizes can be attributed to the fact that their ground states are doubly degenerate which provides for more pathways for faster ground state preparation.

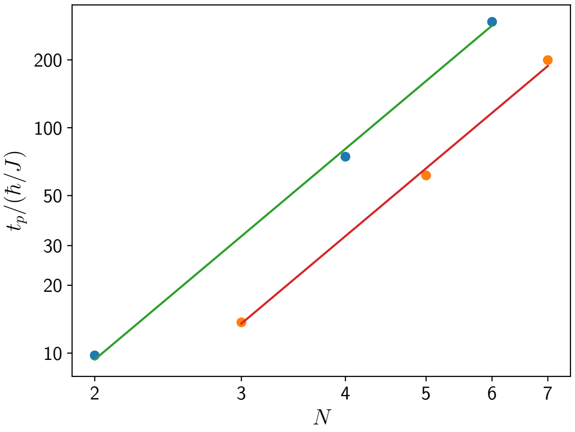

Fig. S4. Scalability of the protocol for the antiferromagnetic Heisenberg model. The preparation time t_p to reach a final dimensionless energy of $\epsilon = 0.2$ grows linearly on a log-log scale, i.e., $t_p \sim N^\alpha$ as in the case of transverse Ising model. The green (N even) and red (N odd) solid lines are the fits to the data with a common exponent α according to $\alpha = 3.11 \pm 0.01$.

Section S5. Entanglement measure for the ground-state cooling of the Heisenberg model

As the ground state of the antiferromagnetic Heisenberg model is highly entangled, this entanglement should be detectable within the cooling dynamics. As an entanglement measure, we use the negativity

$$\mathcal{N}(\rho) = \frac{\left\|\rho^{T_A}\right\|_1 - 1}{2} \tag{S1}$$

where $\|.\|_1$
we first trace out the bath spin and then take half of the remaining system as the subsystem A. Figure S5 shows the negativity of the Ising model and the Heisenberg model as a function of time normalized with respect to the total preparation time t_p. One can clearly see that the steady state of the system exhibits large entanglement for the Heisenberg model, whereas the Ising model is barely entangled. The initial spike in the negativity can be attributed to the fact that typical high-energy states follow a volume law for entanglement measures, while ground states exhibit a weaker area law [51]. Note that this initial entanglement is not useful for quantum information processing tasks [52].

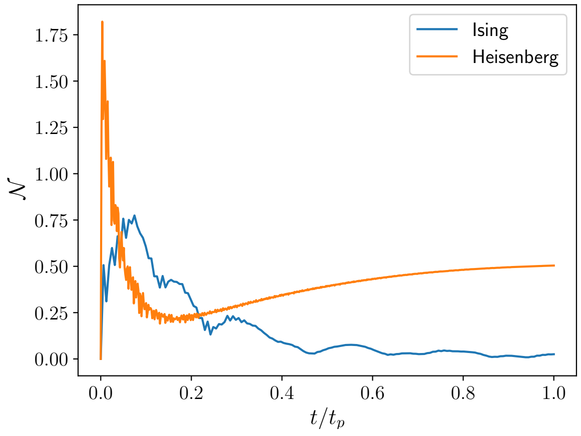

Fig. S5. Negativity as a measure of entanglement of the prepared states in time for a system of $N = 6$ spins. The blue line corresponds to the Ising model ($J/g = 5$) having a low negativity in the long time limit, whereas the orange line corresponds to the antiferromagnetic Heisenberg model with a highly entangled final state. The curves are shown for the optimized parameters for both cases leading to $\epsilon = 0.2$.
